# Status for and associations between disability, employment, barriers for work and health-related quality of life in individuals with multiple sclerosis: A cross-sectional study

**DOI:** 10.1177/20552173261439532

**Published:** 2026-04-23

**Authors:** EC Arntzen, HK Fikke, M Sivertsen, B Normann

**Affiliations:** Faculty of Nursing and Health Sciences, Nord University, Bodø, Norway Department of Physiotherapy, Nordland Hospital Trust, Bodø, Norway; Department of Physiotherapy, Nordland Hospital Trust, Bodø, Norway; Faculty of Nursing and Health Science, Nord University, Bodø, Norway Nordland Hospital trust Department of Physiotherapy Bodø, Norway; Faculty of Nursing and Health Science, Nord University, Bodø, Norway Nordland Hospital trust Department of Physiotherapy Bodø, Norway; Department of Physiotherapy, Nordland Hospital Trust, Bodø, Norway; Department of Physiotherapy, Nordland Hospital Trust, Bodø, Norway

**Keywords:** Multiple sclerosis, EDSS, employment, work, HRQOL, barriers for work, work status

## Abstract

**Background:**

People with multiple sclerosis (pwMS) often face complex challenges leading to work-related barriers, reduced employment and impaired health-related quality of life (HRQOL).

**Objectives:**

Explore status for and associations between disability, employment, work barriers and HRQOL in pwMS.

**Methods:**

Cross-sectional survey, 252 pwMS (49.2% of the 512 invited) aged ≥18 in Nordland County, Norway.

**Measurements:**

Demographics, Expanded Disability Status Scale (EDSS), employment status, MS Work Difficulties Questionnaire-23(MSWDQ-23), and MS Quality of Life-54(MSQOL-54).

**Analysis:**

Descriptive statistics, ANOVA and Pearson's correlation in IBM SPSS-27/28.

**Results:**

Participants had mean EDSS 2.8 [SD2.2], age 52.3 years [SD12.9], 43.7% were currently working, 22.6% held full positions. The mean preferred work percentage in a tailored job was 81.9% [SD24.9] (employed) and 32.3% (non-employed). Disability benefits started early, mean 43.85 years [SD10.65]. Associations were weak between EDSS and work-percentage (*r* = −0.286), work-related barriers (*r* = 0.34) and MSQOL-54 Mental Health Composite Score (HCS) (*r* = −0.27), while moderate for Physical HCS (*r* = 0.53) and between Physical HCS and work percentage (*r* = 0.45).

**Conclusion:**

Unemployment occurred at all disability levels. While physical problems increased over time, mental factors impacted employment at any disability level. However, both employed and unemployed want to work more, indicating possibilities for sustained employment if the job is individually adjusted.

## Introduction

Multiple sclerosis (MS) is a chronic, neurological disease primarily affecting young adults and often leading to a range of physical and cognitive challenges. These challenges can significantly impact employment and health-related quality of life (HRQOL),^1^ resulting in substantial personal and societal burdens.^2,3^ Globally, approximately 61% of the MS population is employed, however, many work part-time.^4,5^ Rates of unemployment and early retirement among people with MS (pwMS) are high but vary considerably across countries, although a slight improvement has been observed since 2010.^6^ While unemployment is most prevalent among individuals with severe disabilities,^1,7^ those with lower disability levels also face barriers to employment, reduced working hours, and job loss.^1,4^ In this group fatigue, impaired balance and walking difficulties are the most common reasons for reduced work participation.^7^ These gradually worsening challenges can significantly affect work ability but are often underestimated in the early phase of MS care.

In Norway, the majority of pwMS have low disability levels,^8^ as measured by the Expanded Disability Status Scale (EDSS).^9^ Nevertheless, Norway reports a workforce exit prevalence of 39%, of pwMS compared to 35.6% across 29 countries, according to a recent meta-analysis.^6^ In contrast, only 10% of the general Norwegian population and 13.1% among individuals living in Nordland county, receives disability pension.^10^ Furthermore, a recent report estimates the total productivity loss from workforce absence and reduced working hours for pwMS at NOK 5.54 billion per year (approximately €485 million).^11^ Similar losses are reported internationally,^12,13^ underscoring the economic impact of insufficient inclusion measures. Factors associated with sustained employment are lower age at MS onset, shorter duration, lower disability level, higher education, light physical work, fewer symptoms related to fatigue, mental health and mobility.^14–16^ Addressing these issues at an early stage may be beneficial for supporting long-term work participation, given the potential of neuro plasticity in the early phases.^17^

Workforce exit is associated with decreased HRQOL.^1^ Fatigue, which is common in pwMS and a major challenge in maintaining employment,^16^ is also reported to significantly reduce HRQOL.^18^ Additionally, socioeconomic status, increased disability, experiences of facing future uncertainty, a sense of loss, anxiety and depression contribute to diminished HRQOL.^16,19,20^ Supporting individual needs at work and in daily life activities is reported to be important for improving HRQOL.^21^ However, knowledge regarding how disability, work and HRQOL are interconnected in pwMS remains limited, particularly among individuals with mild to moderate disability and in northern regions where rural areas dominate and there is a high-risk for MS.^22^ Although a few studies have examined employment in pwMS in Norway, the existing research primarily focuses on incidence, prevalence and estimating the magnitude of economic costs.^2,14,23^ Understanding relationships between disability, work and HRQOL in a unique rural context is essential for developing targeted interventions to support employment and improve HRQOL, thereby reducing societal and economic burdens. This study aimed to provide a comprehensive understanding of employment challenges and HRQOL across disability levels in a rural, high-risk region with the following research question:

“What is the status for and associations between disability, employment, barriers for work and HRQOL in people with MS in Nordland County, Norway?”.

## Materials and method

### Inclusion and exclusion criteria

In this cross-sectional survey all individuals with MS in Nordland county age ≥ 18 years, diagnosed with MS (Mc Donald's criteria),^24^ and registered at the Nordland Hospital Trust (n = 512) were invited to participate. There were no exclusion criteria.

### Outcome measures and procedure

The survey was sent out by mail. The receivers were asked to read the enclosed information letter and invited to sign the informed consent, to answer the survey and return the documents in a pre-paid envelope. The first invitation was sent during the first two weeks of July 2021. A follow-up letter to non-responders including a copy of the questionnaire was sent out in October 2021 and in early January 2022. Participants who returned the questionnaire with some missing data were contacted by phone by HKF and given the opportunity to answer on the phone or to once again receive the survey by mail to fill in the missing items. Data regarding work status, clinical and demographic variables were registered; age, gender, height, weight, social status, type of MS, year diagnosed, EDSS score, occupation and work status (giving the % of a 100% equivalence, disability leave, sick leave and work clearance) as well as education. The participants were also asked how much they would like to work if the job was perfectly adjusted to their needs.

The participants scored two standardized questionnaires; one detecting barriers for work; the MS Work Difficulties Questionnaire-23 (MSWDQ-23)^25,26^ and one examining HRQOL; MS Quality of Life-54 (MSQOL-54).^27^

The MSWDQ-23 is a measure of self-reported workplace difficulties in pwMS. The questionnaire addresses how frequently pwMS perceive psychological/cognitive (11 items)-, physical (8 items)- and external barriers (4 items) related to their current or most recent job and is scored by a 5-point scale (score 0–4, best score 0) with a highest score of 92 (most barriers).^25^ The MSWDQ-23 is translated into Norwegian and validated.^26^

The MSQOL-54 is a multidimensional, valid, HRQOL measure combining generic and MS-specific items in a single instrument.^27^ The questionnaire has 12 subscales: physical function, role limitations-physical, role limitations-emotional, pain, emotional well-being, energy, health perceptions, social function, cognitive function, health distress, overall quality of life and sexual function, as well as two single-item measures: satisfaction with sexual function and change in health. The subscales merge into two health composite scores (HCS) – Physical HCS and Mental HCS, which are the main outcomes. Scores transform linearly to a common 0–100 score range with higher values indicating better quality of life.^27^

### Sample size and setting

We invited all 512 people registered with MS at the Nordland Hospital Trust, the largest hospital in Nordland County, a high-risk area for MS to ensure comprehensive coverage. Nordland county has approximately 244 000 residents distributed across 41 municipalities, most of which are rural. Bodø, the largest city, has around 54 000 inhabitants.

### Statistical analysis

The IBM SPSS version 27/28 was used for the data analysis. Descriptive statistics were used to summarize the demographic, clinical and employment variables, as well as the MSWDQ-23 and the MSQOL-54 scores. Descriptive analyses are reported as means and standard errors for normal distributed data, median, interquartile range and percentage distribution for non-normally distributed data. Pearson's correlation was used to explore associations between variables. The strength of positive or negative correlations was determined using classification from Schober et al. (2018). We conducted one-way ANOVA and Welch ANOVA to examine differences between occupational groups.

## Results

Of the total of 512 pwMS who were invited to participate in the study, 252 (49% of the invited) agreed to take part. The demographic, clinical and employment characteristics of the sample are outlined in Table 1. Within the entire group, 50% were employed the previous year, however, only 43.7% were currently working the previous month. Only 22.6% of those currently working held full positions, and 57.1% had already received disability leave, at a mean age of 43.85 years [SD10.65]. Within our sample, 88% (222 participants) were of working age (≤67 years) [The general retirement age in Norway is 67 years of age.] and had a mean EDSS of 2.6 SD [2.08] which is like the overall group. Among those of working age, 44.1% (98 individuals) did not work the previous year. Among those currently working, the average work percentage the previous month was 59.9% SD [40.87].

**Table 1.** Demographic, clinical and employment status for all participants.

Variable	Mean (SD) or %	Minimum- maximum
Age	52.3 years (12.9)	20–80
Gender: Men, Women	M: 29.8% W: 70.2%	75 men 177 women
Age at diagnosis	38.2 years (11.4)	13–70
Years since diagnosis	13.2 years (10.0)	0–58
EDSS	2.8 (2.2)	0–9
Years since diagnosis	13.2 years (10.0)	0–58 years
Age at diagnosis	39.2 (11.4)	13–70
Employed the previous year	Yes: 50% No: 50%	126 / 126
Currently employed (this month) Yes no	Yes: 43.7% No: 56.3 (missing 2%)	108 / 139
Current work % among employed	56.3% (42.0)	N = 144 0–110
Desired work % if the job was perfectly adjusted (whole group)	53.3% (38.7)	N = 182 0–100
Disability leave	Yes 57.1% No: 41.7%	Yes: 144 No: 105
Sick leave	Yes 8.7% No: 90.1%	Yes: 22 No: 227
Work declearance training	Yes: 3.6% No: 94.4%	Yes: 9 No: 238

Table 2 report barriers for work (MSWDQ-23) and Health Related Quality of Life (MSQOL-54). Barriers for work (MSWDQ-23) averaged 28.6 points SD[18.1] (31% of the maximum score of 92) and were reported across all domains, with higher scores for external barriers, followed by psychological barriers and physical barriers. The most frequently reported barriers included balancing work and home duties, fear of reduced payment, becoming sleepy during task performance and balance problems. HRQOL (MSQOL-54) scores were better for the Mental HCS; 69.7 SD [18.9] than for the Physical HCS; 58.5 SD [19.5] of a maximum score of 100 indicating best HRQOL.

**Table 2.** Descriptive data for the two standardized questionnaires used in the study; the MS work difficulties questionnaire-23 and the MS quality of life-54 for all participants (n = 252).

Variable	Mean (SD) or %	Minimum–maximum
Sum MSWDQ-23 (min 0–max 92)	28.6 (18.1) 31%	0–75
Psychological barriers (11 items) (min 0–max 44)	13.1 (9.2) 30%	0–39
Physical barriers (8 items) min 0–max 32	9.4 (6.8) 29%	0–32
External barriers (4 items) min 0–max 16	6.2 (4.5) 38.8%	0–16
MSQOL Physical min 0–max 100	58.5 (19.5)	13.6–100
MSQOL Mental min 0–max 100	69.7 (18.9)	13.1–100

Tables 3 and 4 show results for those currently working and not working. Working participants had lower mean EDSS (mean 1.8 SD[1.5]) and wished to work 81.9% SD[24.9] if adjustments were made, compared with their current 75.1% SD[30.6]. Non-working participants had higher EDSS (mean 3.72 SD[2.28]) and indicated a desired work-level of 32.3%. The working group had higher educational attainment and reported fewer work-related barriers (mean 22.0 SD[14.7]) versus not employed (mean 34.90 SD[18.74]). The most reported employment barriers were similar across groups.

**Table 3.** Descriptive variables for the split group of participants currently working (mean work % 75.1 (30.6)).

Variable currently working (previous month)	Mean (SD) or %	Minimum–maximum
Age n = 108	47.51 (SD 9.94)	23–71
Gender n = 108	W = 80 M = 28 (74.1%, 25.9%)	
EDSS n = 105	1.72 (1.42)	0–7.5
Age at diagnosis n = 107	37.50 (10.51)	15–64
Years since diagnosis n = 107	9.99 (SD 7.37)	0–37 years
Type of MS n = 108	Relapsing-remitting n = 90, 83.3% Primary progressive n = 9, 8.3% Secondary progressive n = 6, 5.6%	
Social status n = 108	Married n = 51, 47.2% Living with partner n = 25, 23.1% Living alone 32, 29.6%	
Type of work: n = 107	1)Sedentary: n = 39, 36.1% 2)Mixed sedentary/physical: n = 35, 32.4% 3)Physical: n = 30, 27.8 4)Student/homemaker: n = 3, 2.8%	
Current work % (previous month) n = 108	75.11%	0–110
Desired work % n = 75	81.9% (24.9)	20–100
Current disability leave n = 107	Yes: n = 31, 28.7% No: n = 76, 70.4%	
Disability leave % n = 34	52.79 (27.69)	0–100%
Age at disability leave n = 27	41.26 (7.67)	22–61 years
Currently on sick leave % n = 108	Yes n = 11, 10.2% No n = 97, 89.8%	
MSWDQ-23 n = 107	22.00 (14.73)	0–55
Psychological barriers n = 107	10.04 (7.75)	0–35
Physical barriers n = 108	6.89 (5.58)	0–24
External barriers n = 108	5.17 (4.02)	0–14
MSQOL-54 Physical n = 107	68.98 (16.77)	(30.95–100)
MSQOL-54 Mental n = 108	77.06 (16.03)	(36.76–100)

**Table 4.** Descriptive variables for the split group of participants currently not working.

Variable	Mean (SD) or % n = 121	Minimum–maximum
Age		
Gender n = 140	W = 94, M = 46, 67.1%, 32.9%	
EDSS n = 135	3.72 (2.28)	(0–9)
Age at diagnosis n = 140	40.63 (12.01)	(13–70)
Years since diagnosis	15.57 (11.11)	(0–58)
Desired work %	32.32% (33.13)	0–100
Type of MS n = 136	Relapsing remitting: n = 83, 59.3% Primary progressive: n = 25, 17.9% Secondary progressive: n = 28, 20%	
Social status n = 138	Married: n = 62, 44.3% Living with partner: n = 35, 25% Living alone: n = 41, 29.3%	
Type of work n = 125	1)Sedentary: n = 20, 14.3% 2)Combined sedentary/physical: n = 27, 19.3% 3)Physical: n = 74, 52.9% 4)Student/homemaker: n = 4, 2.9%	
Currently on disability leave n = 138	Yes: n = 111, 79.3% No: n = 27, 19.3%	
Disability leave % n = 108	94.63% (18.78)	0–100%
Age at disability leave n = 105	45.00 (10.93)	20–64
Currently on sick leave n = 137	Yes: n = 11, 7.9% No: n = 133, 95.0%	
MSWDQ-23 n = 115	34.90 (18.74)	0–75
Psychological barriers n = 115	15.88 (9.47)	0–39
Physical barriers n = 116	11.88 (7.06)	0–35
External barriers n = 116	7.09 (4.71)	0–16
MSQOL-54 Physical n = 139	50.10 (17.22)	(13.63–93.79)
MSQOL-54 Mental n = 139	64.15 (18.72)	(20.98–97.60)

Correlations between MSWDQ-23 sum score and age were non-significant. There was significant differences in work% (*F* (3, 28.19) = 6.91, *p* = 0.01) between occupational groups: (1) *sedentary/office-based occupations, including managerial roles*; (n = 60, mean 48% SD = 43.82), (2) *mixed physical and non-physical occupations requiring vocational or bachelor-level training* (e.g. nurses, physiotherapists, teachers) (n = 62, mean 44.21%, SD = 44.51), (3) *primarily physically demanding occupations typically not requiring formal higher education* (e.g. hairdressers, carpenters, plumbers) (n = 105, 21.22%, SD = 37.52), (4) *students and homemakers* (n = 7, 23.21%, SD = 36.99). Group 3 had significantly lower work% than 1 and 2 (group 4 had few participants and showed uncertain results). There were, however, no significant differences in barriers for work (sum score of MSWDQ-23) between occupational groups. Levene's test indicated equal variances (*p* = 0.762), and one-way ANOVA demonstrated no significant between-groups effect, F(3, 211) = 1.82, *p* *=* 0.145. The effect size was small (η^2^ = 0.025), indicating that occupational group accounted for only a minor proportion of the variance in MSWDQ-23 scores.

Those who were currently employed reported better HRQOL scores than those not currently employed. Specifically, the employed had a Physical HCS of 69.0 SD[16.8] and a Mental HCS of 77.1 SD[16.03] compared to not employed showing Physical HCS of 50.1 SD[17.3] and a Mental HCS of 64.3 SD[18.7]). There were similar findings for those working the previous year; mean difference 8.19 points (*p* < 0.001) in Physical HCS and 5.66 points (*p* = 0.035) in Mental HCS compared to non-workers, even when adjusting for EDSS. Those working more than 50% reported better Physical (71.51/57.11) and Mental HCS (78.26/68.45) scores than those working less. After controlling for EDSS, higher HRQOL scores remained positively associated with employment (physical HRQOL: partial *r* = 0.216, *p* < 0.001; mental HRQoL: partial *r* = 0.136, *p* = 0.035), meaning that EDSS does not fully explain these relationships, and that physical HRQOL appears more important for maintaining employment.

**Figure 1.**
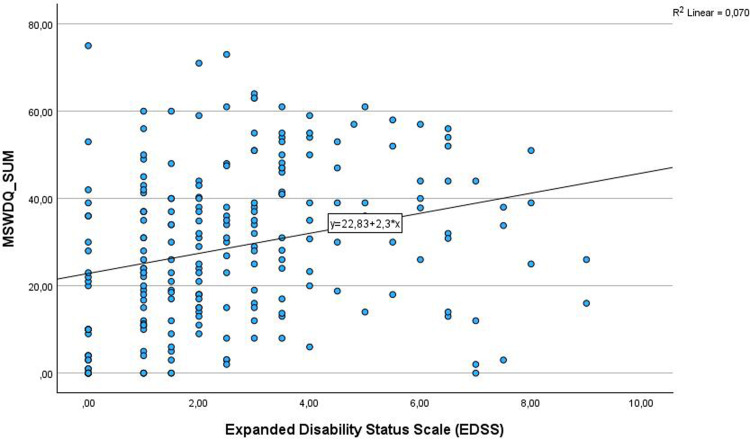
Associations between disability level as measured with the EDSS scale and barriers for work measured by the MS Work Difficulties Questionnaire-23 was low but significant.

**Figure 2.**
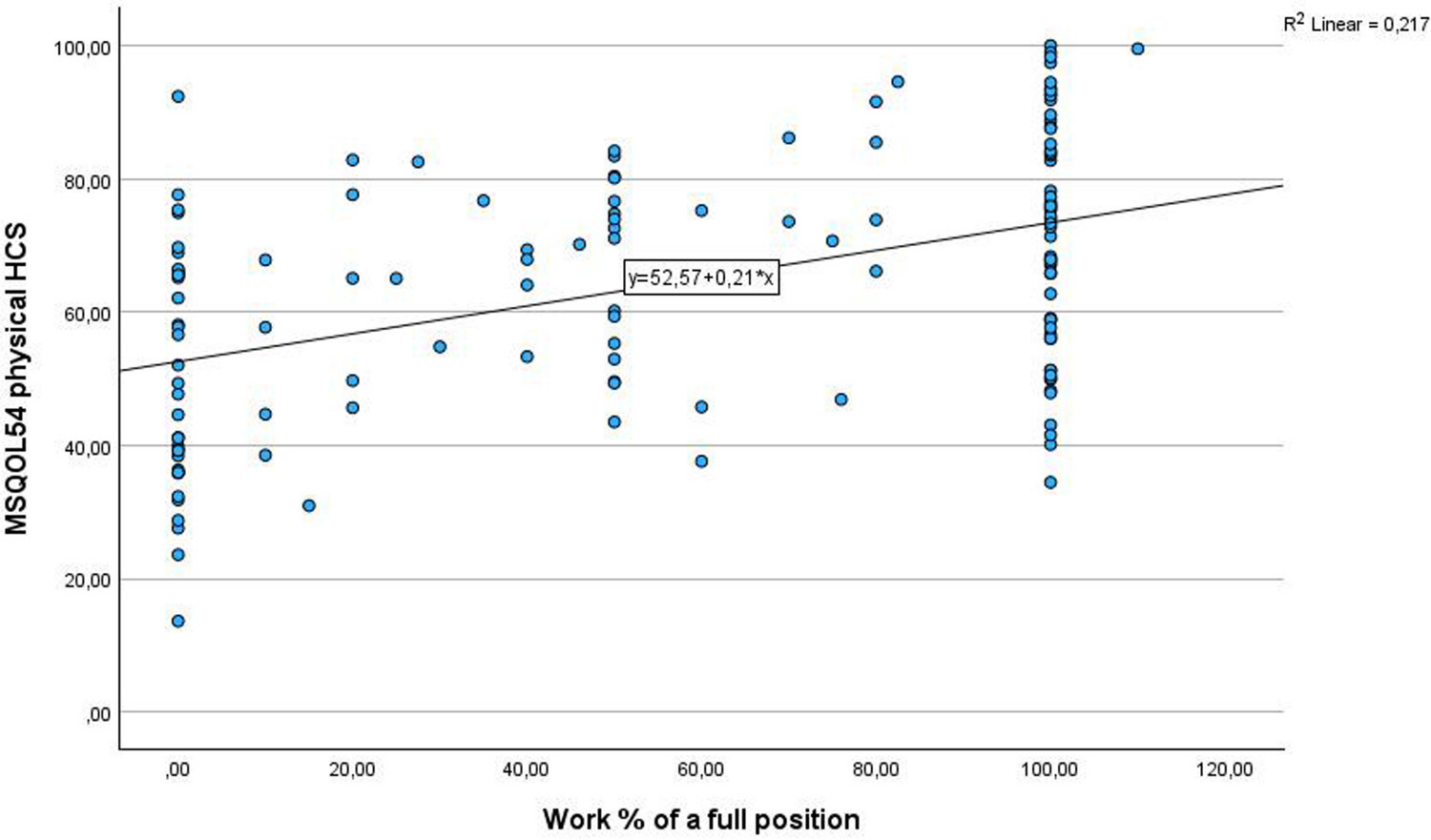
Associations between the Physical Health Composite Score of the Health Related Quality of Life-54 and current work percentage of a full position.

**Figure 3.**
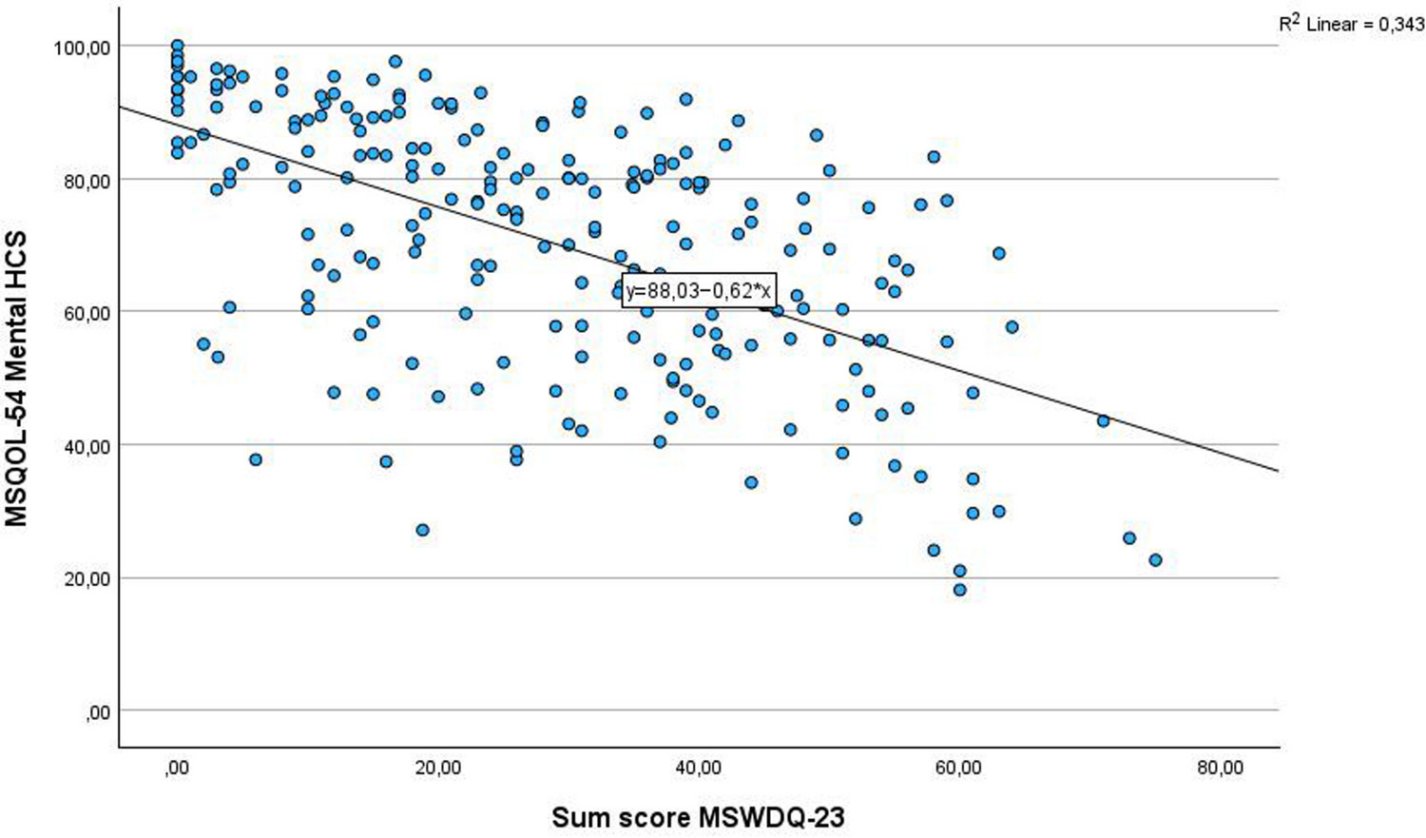
Associations between the Mental Health Composite Score of the Health Related Quality of Life-54 and barriers for work (the MS Work Difficulties Questionnaire-23).

Correlations between variables are shown in Table 5. Overall, disability level (EDSS) had week associations with current work percentage (*r* = −0.286, *p* = 0.001), disability leave percentage (*r* = 0.32, *p* = 0.001), and barriers for work (sum score MSWDQ-23) (*r* = 0.34, *p* = 0.001) (Figure 1). Associations between EDSS and MSQOL-54 Mental HCS were weak (*r* = −0.27, *p* = 0.001), while Physical HCS was moderate (*r* = −0.53, *p* = 0.001). There was a moderate correlation between the Physical HCS and current work percentage (*r* = 0.45, *p* = 0.001) (Figure 2) and a week correlation between Mental HCS and current work percentage (*r* = 0.32, *p* = 0.001). There was a moderate correlation between barriers for work and Physical HCS (*r* = 0.59, *p* = 0.001) and Mental HCS (*r* = 0.59, *p* = 0.001) (Figure 3).

**Table 5.** Correlations between variables calculated by Pearson's correlation.

Variable	EDSS	Current work percentage	Disability leave percentage	Desired work percentage	MSWDQ-23	MSQOL-54 Mental HCS	MSQOL-54 Physical HCS	Years since diagnosis
EDSS	-	*r*= −0.286 **	*r* = 0.32**	*r* = −0.20, non	*r* = 0.34**	*r* = −0.27**	*r* = −0.53**	*r* = 0.34 **
Current work percentage	-	-	-	*r* = 0.66 **	*r*= −0.428**	*r* = 0.32**	*r* = 0.45**	*r*= −0.197 *
Disability leave percentage	-	-	-	*r=* −0.414 **	*r* = 0.224**	*r* = −0.191**	*r* = −0.295 **	*r*= 0.058 non
Desired work percentage	-	-	-	-	*r* = −0.238**	*r* = 0.135 non	*r* = 0.342 **	*r* = −0.338 **
MSWDQ-23	-	-	-	-	-	*r* = 0.59**	*r* = 0.59 **	*r*= −0.083 non
MSQOL-54 Mental HCS	-	-	-	-	-		*r*= −0.48**	*r*= −0.029, non
MSQOL-54 Physical HCS	-	-	-	-	-	-	-	*r* = −0.09, non
Years since diagnosis	-	-	-	-	-	-	-	-

*p < 0.05, **p < 0.01., non = p > 0.05.

Lower Physical HCS was associated with higher barriers for work (MSWDQ-23 scores) when controlling for EDSS (*r* = –0.570, *p* < 0.001), thus, the association is not only about disability status. Moderate associations were observed between Mental HCS and barriers for work *r* = –0.563, *p* < 0.001 meaning that poorer mental health is associated with more work-related difficulties even when disability is controlled for. Associations between Mental HCS and MSWDQ-23 for each of the occupational groups were moderate: group 1 (*r* = 0.528, *p* < 0.001); group 2 (*r* = 0.646, *p* < 0.001); group 3 (*r* = 0.616, *p* < 0.001). Associations between Physical HCS and HRQOL were for group 1 (*r* = 0.605); group 2 (*r* = 0.546, *p* < 0.001); group 3 (*r* = 0.599, *p* < 0.001). In order to see if age was a moderator for these relationships, a linear regression showed a marginal non-significant effect (B = –0.282, *p* = 0.058) and the interaction MSWDQ-23 × age was non-significant (B = 0.007, *p* = 0.086), indicating that age was not a moderator for the associations between work barriers and Mental HCS.

## Discussion

This cross-sectional survey revealed that only 43.7% of the sample were currently employed, despite a relatively young age and moderate disability. Among those working, only 22.6% held full-time positions, which is lower than comparable studies.^6,16^ Notably, both employed and unemployed participants expressed a desire to work more if job adjustments were available, indicating unreleased potential for sustained employment in pwMS. The Mental HCS was higher than the Physical HCS, indicating that physical factors had a greater impact on perceived HRQOL. The moderate association between barriers for work and both Physical and Mental HCS indicates that higher barriers are linked to reduced HRQOL, even when adjusting for disability level.

The disability distribution and EDSS range in the current study mirror those of the Norwegian MS population,^8,14^ suggestion minimal selection bias. Employment levels were also comparable to prior Norwegian,^11,14^ Swedish^28^ and European studies.^4^ However, the 50% response rate may potentially be a limitation. For ethical and privacy reasons, we did not gain any information regarding those who did not answer the survey. Potentially this may have caused bias if, for instance, unemployed individuals had more time to fill out questionnaires or were more interested in filling them out. If so, the rate of unemployment would be inflated even though the range of EDSS was similar to the MS population. The rate of disability pension was markedly higher than in the general Norwegian population, including the northern regions.^10^ This highlights the need for early attention to employment challenges from the time of diagnosis.

As expected, individuals in physically demanding occupations had lower work participation (subgroup 3), consistent with prior research.^16^ However, reported barriers did not significantly differ across occupational groups. Barriers were overall moderate, and their week association with disability level suggests that occupation explained only a minor proportion of the variance in MSWDQ-23 scores. Factors not captured by EDSS or MSWDQ-23, such as depression, anxiety^16,20^ or perceived future deterioration^29^ may also contribute. These findings underscore the multifaceted nature of work participation and reinforce the need for individualized assessments.

Work accommodation was not examined, representing a limitation, particularly since participants expressed willingness to work more with adequate adaptations. Previous qualitative research highlights physical activity at work as a potentially valuable adjustment.^30^ This approach may be particularly relevant considering our findings, where the most common barriers for work were balance problems, fatigue-related sleepiness during tasks and challenges in balancing work and home duties, which are consistent with those reported in previous studies.^5,7,16^ Importantly, many of these difficulties could be alleviated through targeted physiotherapy interventions,^31,32^ physical activity,^33^ exercise and rehabilitation.^34–36^ Such approaches are particularly pertinent given that many pwMS are insufficiently active.^37^ However, implementation is challenging in rural areas with limited access to specialized care, and fewer job alternatives.

Our findings showed a linear association between disability and work barriers only among those with EDSS 0–4, suggesting that barriers should be addressed early, when work adjustments remain feasible. This is aligned with current physical activity recommendations,^38–40^ and should be emphasized within the employment context to promote sustained employment. No association was, however, found among individuals with an EDSS score of 4.5–9. This may highlight that individuals with higher levels of disability are often not working, thus challenges should be addressed earlier, before people leave the workforce.

Those not currently working reported higher barriers across all MSWDQ-23 items. Workers also had significantly higher Physical and Mental HCS compared to non-workers, independent of EDSS level. Physical impairments had a stronger impact on HRQOL than mental factors, which is in contrast with another study indicating that mental factors have a greater impact on HRQOL and work participation than physical factors.^17^ The discrepancy may reflect differences in EDSS levels, the differences in outcome measures used, and contextual factors such as rural Norwegian living conditions, challenging weather, and limited job alternatives, all of which can increase reliance on physical functioning. It may also reflect the traditional focus in MS care, where physical impairments have been given most attention.

Both Physical and Mental HCS were moderately associated with greater work-related barriers (MSWDQ-23 scores), even after adjusting for EDSS, indicating that this association is not solely driven by disability level. Similar patterns were observed within the occupational subgroups. Individuals in the mixed physical and non-physical occupations (group 2) showed the strongest association to Mental HCS, whereas those in the least demanding (group 1) and most physically demanding occupations (group 3) showed the stronger associations between the Physical HCS and work barriers. Group 2 included teachers, nurses and physiotherapists, which require continuous engagement of both mental and physical capacities throughout their workday. The most unexpected result was that individuals in sedentary jobs showed the strongest association between Physical HCS and work barriers. This underscores the importance of addressing physical elements across all job types. Interestingly, disability showed a moderate association with Physical HCS but only a week association with Mental HCS. This may indicate that physical impairments exert a more gradual influence on HRQOL, whereas mental elements may impact HRQOL at any time-point. Since age did not moderate these associations, these findings highlight the need to assess all work-related factors early, regardless of age. Such targeted management of work barriers should be prioritized in future interventions and clinical practice.

The 50% response rate in the current study may be highlighted as a limitation, particularly since we for ethical and privacy reasons, have not gained any information regarding those who did not answer the survey. Potentially this may have caused bias if for instance unemployed individuals had more time to fill out questionnaires or were more interested filling them out, as the rate of unemployment would be inflated even though the variation of EDSS was similar to the annual MS report.

## Conclusion

Unemployment and reduced positions were common among pwMS, even among pwMS having mild disability. Work barriers were moderate, and only weakly associated with disability, suggesting that barriers should be addressed across all EDSS levels. Physical HRQOL was lower than mental, and the association was moderate between EDSS and Physical HCS while weak for the Mental HCS. However, the association between barriers for work and both physical and mental HRQOL was moderate, emphasizing the importance of addressing barriers. Importantly, many participants expressed a desire to work more if suitable adjustments were available, highlighting an opportunity for sustainable employment. Early identification and targeted management of work barriers should therefore be prioritized in future interventions and clinical trials.
